# Report of the Diseases of Edinburgh for September, 1808

**Published:** 1808-11

**Authors:** John Roberton

**Affiliations:** Surgeon


### *Report of the Diseases of Edinburgh for September,* 1808.

By John Roberton, Surgeon.

This month commenced with rain, which, though by no means heavy, continued for the most part for the first three or four days. The weather then became variable till about the middle of the month, when we had six or seven days of delightful weather. It again became variable, and in this way the month terminated. Toward the end of it we had some frosty mornings.

The equinoctial gales, as they are called, which are usually pretty severe with us, have not been so violent as on former occasions. These hurricanes are often very dreadful in this part of the world, so much so, that it is with the greatest difficulty that either foot passengers or carriages of any sort can make their way along the streets. During these winds, accidents occur from the frequent falling of pans from the chimney tops, and even chimney tops themselves.

The thermometer, during the whole of the month, stood, for the most part, about 60°; sometimes indeed it was as high as 70°, but that was seldom. The barometer varied considerably during the whole month.

Diarrhœa and cholera, formerly mentioned, have not abated in severity; indeed, in some instances, they have been uncommonly severe, holding at defiance, at least for a considerable time, every remedy that could be thought of for their removal. Among the most fortunate applications, tonics, in conjunction with antispasmodics, seem the most effectual in lessening their severity, and at length in entirely removing these complaints. It is absolutely necessary too, now that the weather, particularly in the evenings, begins to be cold and disagreeable, that great care should be taken, especially by those affected with these disorders, to protect the body against its effects. For this purpose, in this climate, our winter clothing ought not to be too long delayed. Those therefore in the habit of wearing flannel, should now gradually accustom themselves to its use; perhaps, thick cotton cloth, worn next the skin, may answer all the good purposes of the former, and be at all times a much cleaner dress. As the protecting of the feet against dampness has always been considered of the utmost importance, while cork soles, fur lining for the shoes, &c. and socks of various kinds, have been used, a more simple, and in my opinion, a more effectual preventive has been entirely overlooked. I allude to the use of cloth slippers over the stockings. If shoes be worn, these [slippers]

pers] may be exactly fitted to the foot, and not permitted to extend above the edge of the shoe; if boots be worn, they may be of sufficieut length to cover the ancle joint. Thro' this substance no moisture can pass; and even if it should penetrate the shoe or the boot, it cannot reach the stocking immediately covering the foot. This is a habit I strenuously recommend, having witnessed its beneficial effects in numerous instances.

Intermittent fever has lately been more frequent here than we recollect it to have been for many years. The bark having long maintained its superiority in such affections, is of course the most common remedy we have recourse to. Such has been the obstinacy of some of the cases of this disease which have lately occurred amongst us, that we have found it absolutely necessary, to prevent a recurrence of the paroxysm, to continue the bark for a considerable number of days after their disappearance. By inattention to this rule, we have often had the disease protracted for a much longer time than it would otherwise have been. Such a degree of obstinacy in this disease has seldom been met with in this city.

Scarlatina and typhus fever have likewise prevailed, though not with great severity, and least so the former. The latter, indeed, has been various in its effects, according to the part of the city, or the situation of life of the persons affected by it. I have uniformly observed, that all epidemic diseases, when they occur in the immediate neighbourhood of filth or putrefying substances of any sort, are always most destructive to the inhabitants; and in proportion as the people are more cleanly, and further removed from such effluvia, they enjoy better health. The very causes of such complaints indicate the best method of cure, viz. instant removal from such sources of contamination.

The Small-pox and the Measles have again appeared in various parts of this city, and, in some instances, the former of these has been of the confluent kind. Why should the obstinacy and perverseness of individuals *still* be permitted to propagate such a loathsome disease amongst us? Certainly, as I have repeatedly urged, a well regulated internal police of every city and town in our island, would soon extirpate that destructive pest.

Several cases of Dysentery have lately appeared in this city, but the complaint has not been universal.

*St. James's Street.*

